# Dynamics of PO_2_ and VO_2_ in resting and contracting rat spinotrapezius muscle

**DOI:** 10.3389/fphys.2023.1172834

**Published:** 2023-07-19

**Authors:** Aleksander S. Golub, Bjorn K. Song, William H. Nugent, Roland N. Pittman

**Affiliations:** ^1^ Department of Physiology and Biophysics, Medical College of Virginia Campus, Virginia Commonwealth University, Richmond, VA, United States; ^2^ Song Biotechnologies LLC, Cockeysville, MD, United States

**Keywords:** oxygen consumption, oxygen supply, oxygen supply-demand matching, microcirculation, muscle contraction

## Abstract

This study examined changes in interstitial PO_2_, which allowed calculation of VO_2_ during periods of rest, muscle contraction and recovery using an *in situ* rat spinotrapezius muscle preparation. The PO_2_ was measured using phosphorescence quenching microscopy and the muscle VO_2_ was calculated as the rate of O_2_ disappearance during brief periods of muscle compression to stop blood flow with a supra-systolic pressure. The PO_2_ and VO_2_ measurements were made during “5 s compression and 15 s recovery” (CR) cycles. With all three stimulation frequencies, 1, 2 and 4 Hz, the fall in interstitial PO_2_ and rise in VO_2_ from resting values occurred within the first 20 s of contraction. The PO_2_ during contraction became lower as stimulation frequency increased from 1 to 4 Hz. VO_2_ was higher at 2 Hz than at 1 Hz contraction. With cessation of stimulation, PO_2_ began increasing exponentially towards baseline values. After 1 and 2 Hz contraction, the fall in muscle VO_2_ was delayed by one CR cycle and then exponentially decreased towards resting values. After 4 Hz stimulation, VO_2_ increased for 2 cycles and then decreased. The post-contraction transients of PO_2_ and VO_2_ were not synchronous and had different time constants. With further analysis two distinct functional responses were identified across all stimulation frequencies having PO_2_ during contraction above or below 30 mmHg. The corresponding VO_2_ responses were different - for “high” PO_2_, muscle VO_2_ reached high levels, while for the “low” PO_2_ data set muscle VO_2_ remained low. Recovery patterns were similar to those described above. In summary, local microscopic PO_2_ and VO_2_ were measured in resting and contracting muscle *in situ* and the post-contraction transients of PO_2_ and VO_2_ were all much slower than the onset transients.

## Introduction

The ability of the microcirculation to provide an adequate oxygen supply over a wide range of metabolic activity represents a remarkable physiological phenomenon (Poole and Jones, 2012; Koga et al., 2014). The nature of mechanisms synchronizing and matching the O_2_ delivery and consumption rates have been under investigation for more than a century (Roy and Brown, 1880; Roy and Sherrington, 1890; Krogh, 1919; Krogh, 1922; Rowell, 2004; Clifford, 2007; Clifford and Tschakovsky, 2008; Tschakovsky and Joyner, 2008; Sarelius and Pohl, 2010; Clifford, 2011; Golub and Pittman, 2013a; Golub and Pittman, 2013b; Joyner and Casey, 2015). It is assumed that the basic principles of local blood flow regulation are the same for all organs; however, skeletal muscle is the most appropriate organ for experimental studies due to the controllable and measurable functional activity. Metabolic rate and O_2_ consumption in muscle can be changed over a wide range by voluntary control of workload intensity or by electrical stimulation. This opportunity opens an experimental window into the general mechanisms of coordination between O_2_ demand and supply in organs and tissues.

Studies of skeletal muscle at transitions from rest to steady exercise and then back to the resting state have a long history of achievements, being thoroughly discussed in reviews, covering thousands of sources (Borsheim and Bahr, 2003; Joyner and Wilkins, 2007; Wagner, 2011a; Casey and Joyner, 2011; Poole and Jones, 2012; Joyner and Casey, 2015). The main methodological approach for studying VO_2_ in a muscle is Fick’s principle representing mass conservation for oxygen in blood (Weibel, 1984). In a steady state of O_2_ delivery and consumption, the oxygen flux from capillaries to cells in an organ is equal to the product of blood flow and arterio-venous difference of blood oxygen content, i.e., the oxygen extraction. At the onset of muscle contraction and activation of oxidative metabolism, an additional amount of oxygen can be delivered due to increased O_2_ extraction at constant blood flow, increased blood flow at constant oxygen extraction and the augmentation in values of both these variables. The interaction of individual factors that determine the oxygen flux to the parenchymal cell and limit the maximum oxygen consumption is demonstrated in Wagner’s diagram representing the graphical solution of two pertinent equations describing Fick’s principle and Fick’s law of diffusion (Wagner, 1996; Wagner, 2011b; Spurway et al., 2012). Increased oxygen extraction from blood leads to a decrease in the mid-capillary PO_2_, which reduces the diffusive flux of oxygen and limits oxygen consumption by muscle fibers. Thus, a balance between convective and diffusive transport is achieved at the intersection of the two “Fick” lines. An important assumption in this analysis was having zero PO_2_ at the mitochondria, which was reasonable for the analysis of factors limiting the maximal O_2_ consumption. Moreover, in the case of a limited metabolic capacity of mitochondria, the maximal VO_2_ could be limited by this factor (Wagner, 2011b).

The experimentally established dependence of VO_2_ on the PO_2_ for muscle tissue and mitochondria under “physiological conditions” (Golub and Pittman, 2012; Wilson et al., 2012; Golub et al., 2018) formed the basis for a graphical analysis of the O_2_ delivery and consumption in the range between resting and maximal workload rate. For that purpose, the experimental dependence of VO_2_ on interstitial PO_2_ has to be measured on a microscopic scale, throughout the entire range of functional activity in the muscle. The narrow interstitial space between the capillaries and muscle fibers is the interface between the O_2_ delivery and consumption part ofthe muscle. The compartment is located between capillaries and myocytes, thus the interstitial PO_2_ can be used as a sensitive indicator of the balance between rates of O_2_ delivery and consumption in the muscle. The VO_2_(PO_2_) dependence curves for muscle cells are determined by two factors: mitochondrial respiration and intracellular diffusion (Golub and Pittman, 2012). Thus, a set of curves VO_2_(PO_2_) for various rates of workload characterizes the entire function of O_2_ consumption in muscle cells.

The development of phosphorescence quenching technology (Vanderkooi et al., 1987; Vanderkooi et al., 1991) has allowed measuring oxygen-related variables in resting and contracting muscles on the microscopic scale (Behnke et al., 2001; McDonough et al., 2001). The successful combination of *in vivo* microscopy in a thin skeletal muscle and the phosphorescence quenching method was employed for recording the transients in microvascular PO_2_ together with blood flow velocities and RBC flux (Behnke et al., 2002; Behnke et al., 2003a; Behnke et al., 2003b). We have developed the compression technique, combined with phosphorescence quenching microscopy, to enable a direct determination of VO_2_ in microscopic volumes of muscle during rest, contraction and recovery (Roy and Brown, 1880; Smith et al., 2002; Golub et al., 2011; Golub and Pittman, 2012; Nugent et al., 2016a).

We have applied that technique to collect a set of oxygen dependence curves in rat spinotrapezius muscle at different levels of metabolic activity (Golub et al., 2018). As a result of this work, a model of balance between O_2_ delivery and consumption at different workloads in the muscle was developed.

This methodology opens up the possibility of directly and simultaneously measuring oxygen tension on the surface of myocytes and the rate of their respiration in various states of muscle function: rest, work and recovery. There is a belief that in a resting muscle there is a balance between the rate of oxygen delivery to the interstitium and oxygen consumption by myocytes. During the transition from rest to work, the increased oxygen consumption by the muscle reduces interstitial PO_2_ and thus increases the transmural flow of oxygen from the capillaries. This mode of regulation has its limitations so that the component of convective transport may require an active vascular response. In addition, mechanical interference with perfusion occurs in a contracting muscle. The same complex interactions also occur during the transition from work to rest, so that there is a restoration of muscle resources used to cover the energy imbalance during work. The balance of oxygen delivery and consumption results from the interaction of many factors. The imbalance of oxygen demand and supply is the subject of this experimental study.

The experimental study of transients following the onset and offset of muscle contraction at different intensities opens a window into understanding the functional mechanisms by which muscle cells interact with the microvascular network (Hill et al., 1924; McDonough et al., 2001; Behnke et al., 2002; Borsheim and Bahr, 2003; Poole and Jones, 2012). Over recent decades the study of these transients on a microscopic scale, at which the musculature and vasculature interact, has yielded significant progress in this area (Behnke et al., 2001; McDonough et al., 2001; Behnke et al., 2002; Kindig et al., 2003). Investigations of the regulation of oxygen consumption are focused on the issue of determining these factors, namely, the role of regulation of oxygen delivery *versus* cellular metabolic control (Poole and Jones, 2012). In the analysis of our experimental results, we assumed that oxygen delivery and consumption in contracting muscle tissue are well-coordinated and interrelated, hence forming an integrated O_2_ processing system.

## Materials and methods

### PO_2_ and VO_2_ monitoring in skeletal muscle *in situ*

**FIGURE 1:**
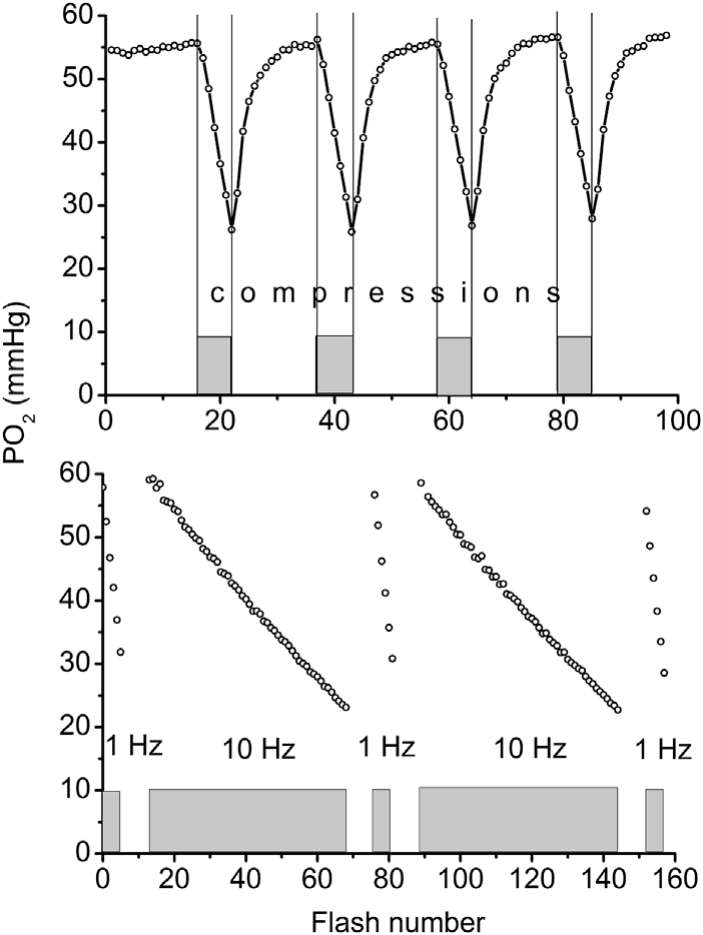
**Top:** Example of continuous measurement of interstitial PO_2_ in the rat spinotrapezius muscle with a flash rate of 1 Hz. Compression air pressure of 140 mmHg from automatic pressure controller was quickly applied via thin film airbag to muscle for 5 s time period (grey rectangles and vertical lines). During 5 s of compression the PO_2_ fall was recorded, then the airbag pressure was momentarily reduced to a low level of 5 mmHg and interstitial PO_2_ in muscle was restored to its initial level during the next 15 s. The rate of oxygen disappearance (initial slope for 5 s line segments) was converted to VO_2_ (Nugent et al., 2016a). Measuring compression-recovery (CR) cycle is properly adjusted for VO_2_ measurements with PQM technique. **Bottom:** Principle of determination of oxygen photo-consumption by the method in muscle. The PO_2_ was recorded during the 5 s intervals of muscle compressions, with intermittent excitation flash rates of 1 and 10 Hz (five 5 s compression time intervals are shown as gray boxes). Excitation flash light source was synchronized with the air bag pressure controller, so that the longer periods of pressure release were not recorded in this test. Linear fitting of these data returns the values of oxygen disappearance rates, relevant for the calculation of oxygen consumption by the method (Nugent et al., 2016a).

The compression of a thin muscle using a transparent airbag, rapidly inflated to a supra-systolic pressure (140 mmHg) was employed for measuring the rate of O_2_ disappearance from the interstitium due to tissue respiration (Golub et al., 2011; Nugent et al., 2016a). The PO_2_ decrease in the interstitial space, loaded with a phosphorescent oxygen probe, was recorded using phosphorescence quenching microscopy (PQM). Analysis of the O_2_ disappearance curves (ODC) yielded the local rate of oxygen consumption, VO_2_, and established its dependence on PO_2_ for skeletal muscle *in situ* (Golub et al., 2011; Golub and Pittman, 2012; Nugent et al., 2016b). The VO_2_ in muscle fibers was calculated from the initial slope of the ODC at the onset of muscle compression, and O_2_ solubility in muscle. The method required a brief compression to avoid a shortage of oxygen. After rapid release of airbag pressure, the PO_2_ was quickly restored to the baseline value. The entire muscle on the thermostated pedestal of the animal platform was compressed. The total time period for interstitial PO_2_ measurement represented a complete muscle compression and recovery (CR) cycle, which could be repeated many times without significant effect on the studied variables (Nugent et al., 2016a). A 20-s CR cycle, consisting of 5 s of muscle compression and 15 s free perfusion (Figure 1, top), provided a reliable measurement of VO_2_ in resting and contracting muscle (Nugent et al., 2016a; Nugent et al., 2016b).

### Oxygen consumption by the method

The PO_2_ sampling frequency for serial VO_2_ measurements also has limitations (Golub et al., 2011; Golub and Pittman, 2012; Nugent et al., 2016a). The interstitial oxygen tension in a muscle was measured with a flash rate of 1 Hz. The oxygen consumption by the phosphorescence quenching method itself was estimated by comparison of measurements obtained at flash rates of 1 and 10 Hz at the same tissue site (Figure 1, bottom) (Nugent et al., 2016a). The fraction of oxygen consumed by the method was (6.9 ± 0.5) 10^−3^, and was determined at 56 sites in the rat spinotrapezius muscle. This meant that the PO_2_ measuring procedure itself reduced the PO_2_ by 0.7% per flash, and it is not accumulated due to the 1-s long time interval between two consecutive excitation light pulses. The excitation energy density and oxygen probe concentration were adjusted so that the VO_2_ by the method would not exceed a 1% reduction of the ambient PO_2_ at the measurement site.

### Animal experiments

The following animal protocols and experimental procedures were approved by the Virginia Commonwealth University Institutional Animal Care and Use Committee and are consistent with the National Institutes of Health Guidelines for the Humane Treatment of Laboratory Animals, as well as the American Physiological Society’s Guiding Principles in the Care and Use of Animals. Briefly, 15 male Sprague-Dawley rats (BW = 325 ± 15 g; Harlan, Indianapolis, IN) were given a pre-operative intraperitoneal dose of Ketastet and Acepromazine (75 mg/kg and 2.5 mg/kg, respectively) to establish a sufficient plane of anesthesia for incision and cannulation procedures. The femoral vein was then accessed and cannulated with polyethylene tubing (PE-90) to enable the continuous infusion of alfaxalone acetate (Alphaxan, Vetoquinol UK Limited, Buckingham, MK18 1 PA; ∼0.1 mg/kg/min), which maintained, with responsive adjustment to animal reflexes, heart rate and oxygen saturation indicators, a steady plane of anesthesia through the conclusion of surgical preparation and measurements. A tracheal cannula of PE-240 tubing was inserted to maintain a patent airway. Animal anesthesia status was monitored with a veterinary pulse oximeter (PulseSense VET, www.nonin.com). The SpSO_2_ probe was placed on the hairless, left hindpaw, and the left hindlimb was in contact with the heating pad of the animal platform, with the expectation of good perfusion of the skin in the foot containing mostly arterial blood. Heart rate was 303 ± 4 (65) min^-1^ and oxygen saturation was 88 ± 1 (65) %, below the normal range and not used to calculate arterial PO_2_. Following the completion of experimental measurements, animals were euthanized with an overdose of Euthasol (150 mg/kg i. v., pentobarbital component; Delmarva; Midlothian, VA).

### Rat spinotrapezius muscle preparation

Surgical preparation of the rat spinotrapezius muscle was similar to the original descriptions (Gray, 1973; Engelson et al., 1986; Schmid-Schonbein et al., 1986; Bailey et al., 2000). The muscle was placed on a trans-illuminated pedestal of the animal platform, thermo-stabilized at 37°C (Golub and Pittman, 2003). In order to minimize muscle movement for isometric contractions, the edges of the muscle were fixed with 10–12 sutures to a rigid frame (Bailey et al., 2000). For the purpose of electrical stimulation, two chlorided silver wire electrodes were attached along the side edges of the muscle preparation. In order to ensure proper muscle fixation and electrode connection, a short electrical stimulation (1–5 s) was applied at the end of the preparation period. The muscle was allowed to stabilize for about 20 min while the phosphorescent probe was loaded into the interstitium (Golub et al., 2011). The muscle was then covered with a polyvinylidene chloride gas barrier film (Krehalon, CB-100; Kureha, Japan). An objective-mounted airbag made of the same film provided muscle compression at a supra-systolic pressure (Golub et al., 2011; Golub and Pittman, 2012). The airbag was pneumatically connected to a custom-built air pressure controller containing a diaphragm air pump (LT24, www.pentairaes.com), an electro-pneumatic regulator T3220 (www.marshbellofram.com) and a cycle timer, set for a 5 s period of high pressure at 140 mmHg and 15 s of low pressure at 5 mmHg. The low pressure in the airbag allowed free blood circulation, while providing a tight contact of the gas barrier film to the muscle surface.

### Intravital and phosphorescence quenching microscopy

**FIGURE 2:**
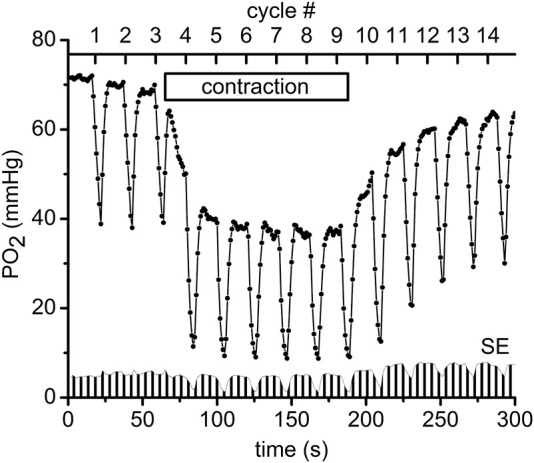
An example of the experimental procedure performed in muscle regions that consisted of measuring the interstitial PO_2_ every second for a time period of 300 s. Every 20 s a pulse of supra-systolic air pressure momentarily compressed the muscle for 5 s and then reduced the pressure to 5 mmHg for recovery during the next 15 s. The initial slope of the saw teeth indicates the rate of oxygen disappearance required to calculate VO_2_ (Nugent et al., 2016a). The scale on the top shows the numbers (#1–14) of the CR cycles (20 s each). Immediately after the end of compression #3 the electrical stimulation of the muscle started at 1 Hz and lasted until the end of CR#9 compression. The “contraction” box represents the electrical stimulation time interval. In total 11 experimental PO_2_ time courses, recorded in different microscopic regions of the same muscle preparation, were averaged and plotted as mean PO_2_’s in this diagram. Standard errors (SE) are presented as a fence-like pattern along the abscissa for convenience.

Measurements of PO_2_ and VO_2_ were carried out using an Axioimager-2m microscope with a 20X/0.8 Plan-Apochromat objective lens (Carl Zeiss, Germany). The measurement technique has been described in detail in our previous publications (Golub et al., 2011; Golub and Pittman, 2012; Nugent et al., 2016a; Nugent et al., 2016b), except for the application in the current work of the Oxyphor R2 dendrimer phosphorescent probe (www.oxygenent.net) whose calibration parameters were taken from the manufacturer (Lo et al., 1996). This R2 probe was chosen for its relatively low molecular weight (2.7 kD) and water-solubility that facilitated its loading into the interstitial space of a thin muscle by topical application of a 10 mg/mL solution for 30 min to the surgically exposed tissue. Octagonal regions of 300 µm diameter, containing no large vessels and separated by about 1 mm from each other were selected for PO_2_ and VO_2_ measurement sites in the central region of the muscle. PO_2_ was sampled at 1 Hz during 300 s of PO_2_ data collection (Figure 2). A color video camera KP-D20B (www.hitachikokusai.com) was employed for imaging and selection of the measurement sites.

### Experimental protocol

The following sequence of experimental events (at each site) as presented in Figure 2 (Behnke et al., 2009; Poole and Jones, 2012): baseline interstitial PO_2_ and VO_2_ were measured at rest during a 1-min time interval, then during 2 min of isometric contraction induced by electrical stimulation (10 V and 20 m duration) and finally during a 2-min post-contraction recovery period. In order to obtain data for different exercise intensities, 1, 2 and 4 Hz frequencies of electrical stimulation were used in different microscopic regions of muscle; the corresponding duty cycles were 20 m, 40 m and 80 m, respectively. For convenience the time scale of the experimental procedure was presented as CR cycle numbers (#, presented in the upper scale of Figure 2). The automatic cycling of a series of 14 tissue compressions started with 3 cycles of compression/release in the resting muscle. Then, the electrical stimulation started after the pressure release at CR cycle #3, so that the muscle was contracting during CR cycles #4–9 (Figure 2). Immediately after decompression in CR cycle #9, the stimulation stopped and the PO_2_ was recorded during the post-contraction CR cycles #10–14, as shown in Figure 2. Depending on preparation stability, the PO_2_ time course data were collected from 5 to 11 different sites in the central region of the same muscle. The time interval between the end of one 300 s record at one site on the muscle and the beginning of measurements at the next site was about 7.5 min (453 ± 14 s; N = 105).

### Statistics

The Levenberg-Marquardt algorithm was used for PO_2_ calculations to fit the multiple phosphorescence decays with codes made using LabView software (www.ni.com). Statistical calculations and parameter fitting were made with the OriginPro 8.1 (originlab.com) software package. All data are presented as Mean ± SE (N, number of measurements).

## Results

### Stimulation rates of 1, 2 and 4 Hz

**FIGURE 3:**
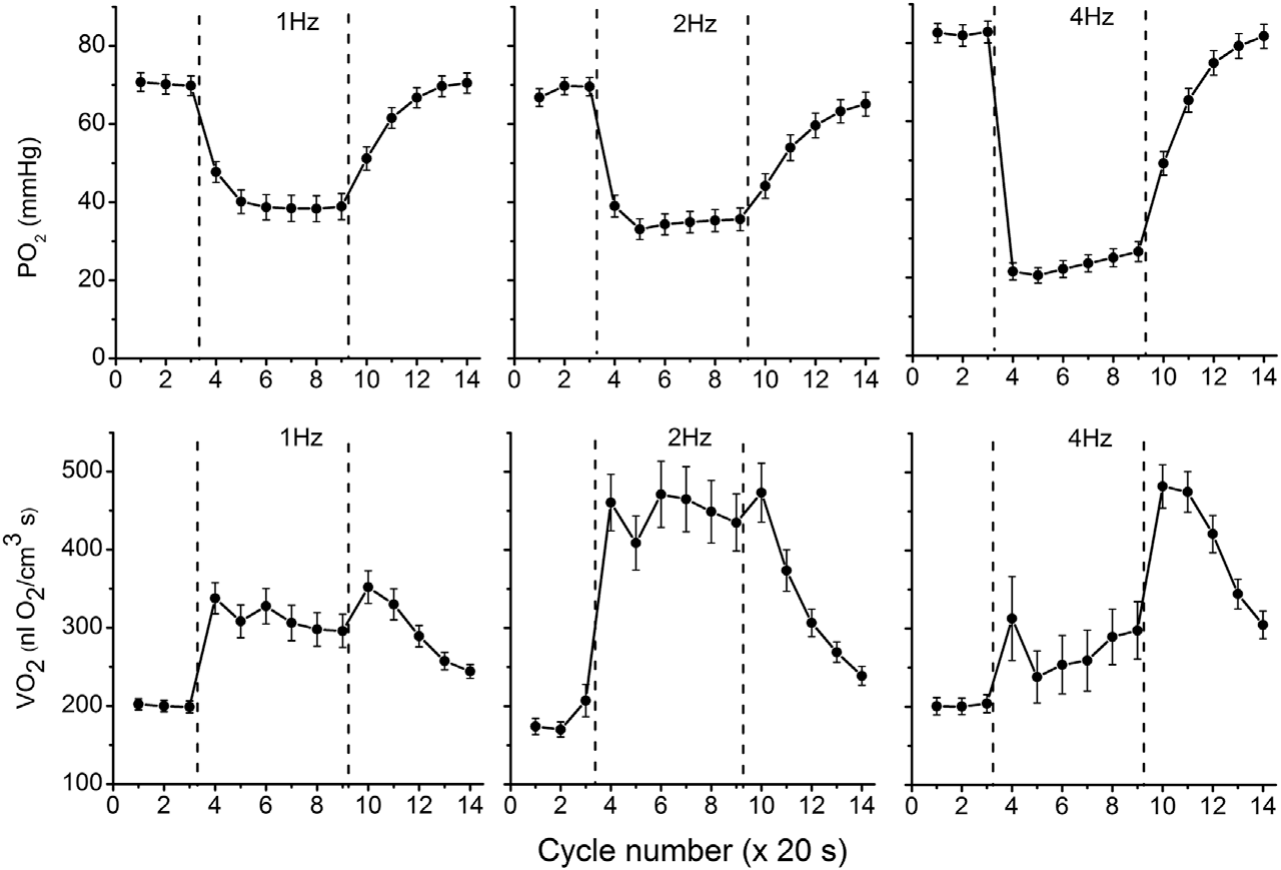
Interstitial oxygen tension (PO_2_, mmHg) and rate of oxygen consumption (VO_2_, nl O_2_/cm^3^ s), recorded in microscopic volumes of rat spinotrapezius muscle in response to electrical stimulation at 1, 2 and 4 Hz. The time unit is the number of CR cycles (20 s each). The electrical stimulation of the muscle started immediately after the end of CR #3 and stopped at the end of CR #9; the contraction time interval is marked by vertical dashed lines. The baseline (rest, CR cycles #1–3) is followed by the transition from rest to contraction (CR cycles #3–5), steady state contraction (# 5–9) and then the post-contraction recovery transients (CR cycles #9–14). Averaged PO_2_ and VO_2_ for segments of experimental curves and their numbers are presented in Table 1.

The data on interstitial PO_2_ and muscle tissue VO_2_ in experiments with 1, 2 and 4 Hz stimulation are presented in Figure 3 and Table 1. In resting muscle, the mean PO_2_ and VO_2_ were similar for all frequencies of stimulation, with the exception of pre-contraction PO_2_ = 82 mmHg in the 4 Hz series, which is 12 mmHg higher (*p* < 0.01) than baseline (CR cycles #1–3) PO_2_ for 1 and 2 Hz contraction. The transition of VO_2_ to its “steady state” contraction (CR cycles #3–5) in response to stimulation happened within a single CR cycle. The fall of PO_2_ to a stable low level took three, two and one cycle at 1, 2 and 4 Hz stimulation rates, respectively. The PO_2_ during the steady contraction period (CR cycles #5–9) decreased between 1 and 4 Hz stimulation rates, with a decrement of about 5 mmHg per Hz (Table 1). Compared with the resting state, the VO_2_ at 1 Hz stimulation (CR cycles #5–9) increased by a factor of 1.54, at 2 Hz by a factor of 2.42, but the VO_2_ at 4 Hz contraction was only slightly higher than at rest, by a factor of 1.32.

**TABLE 1:** Experimental data on interstitial PO_2_ and muscle VO_2_ at rest and during “steady-state” contraction and time constants for the recovery transients for stimulation rates 1, 2 and 4 Hz.

--	Rest		Contraction		Recovery	
Contraction rate, (number)	(#1–3) PO_2_ mmHg	(#1–3) VO_2_ nl O_2_/cm^3^s	(#5–9) PO_2_ mmHg	(#5–9) VO_2_ nl O_2_/cm^3^s	(#10–14) PO_2_ τ, s	(#10–14) VO_2_ τ, s
1 Hz, (41)	70 ± 1	200 ± 4	39 ± 1	307 ± 10	40	43
2 Hz, (38)	69 ± 1	184 ± 8	35 ± 1	446 ± 18	38	46
4 Hz, (26)	82 ± 2	202 ± 6	24 ± 1	267 ± 16	40	*

Data are presented as Mean ± SE. The number of averaged experimental curves, i.e., recorded PO_2_ and VO_2_ time courses in individual microscopic regions of muscle, are presented in parentheses after the stimulation frequency. Transient marked (*) could not be fitted with a monoexponential function.

Each of experimental states of the muscle (rest, contraction and recovery) contains results of several CR, measurement cycles. The PO_2_ and VO_2_ for rest (CR cycles #1–3) and “steady state” contraction (# 5–9) are combined in groups and averaged; thus, the total number of data points in these groups has to be multiplied by 3 and 5, respectively. The last two columns show the time constants for an exponential fit of post-contraction transients of PO_2_ and VO_2_ (CR, cycles #10–14).

With cessation of muscle stimulation, after CR cycle #9, the PO_2_ began to increase exponentially towards pre-stimulation values. The PO_2_ rise had a time constant of 38–40 s (Figure 3; Table 1). After the end of stimulation, the high level VO_2_ was extended for at least one more CR cycle. In the experiments with 4 Hz stimulation, muscle VO_2_ first increased to a maximum for 2 CR cycles and then began to decrease toward the resting values. Thus, the post-contraction transients of PO_2_ and VO_2_ did not start synchronously and had different time constants (Table 1; Figure 3).

### Two patterns of functional response

**FIGURE 4:**
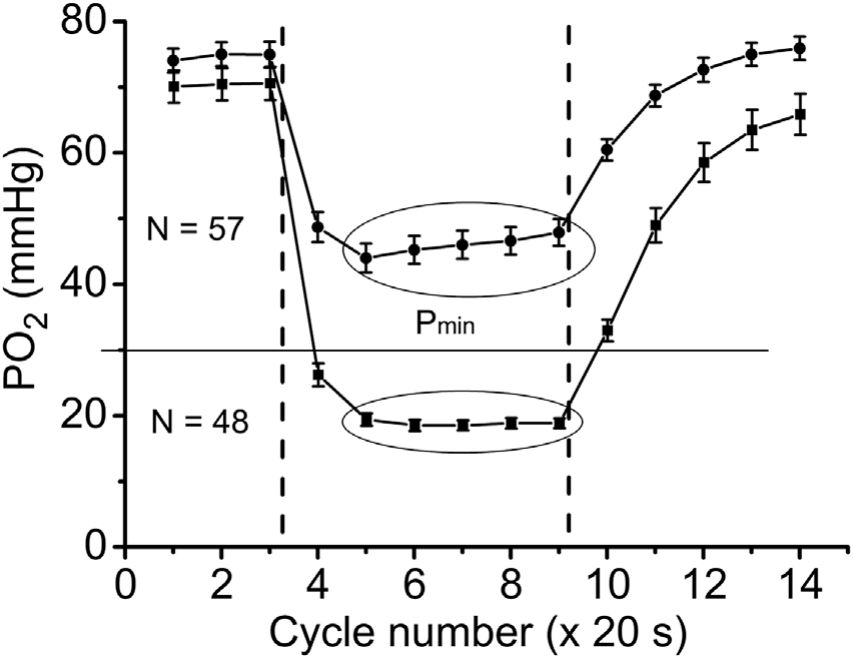
Time courses of interstitial PO_2_ in response to muscle contraction. All experimental data were sorted out according to the PO_2_ during “steady-state” contraction (“low workload group,” P_min_ > 30 and “high workload group,” P_min_ <30 mmHg). Low workload group contained all 1 Hz stimulation data and part of data with 2 Hz stimulation. High workload group contained all 4 Hz data and part of the 2 Hz group. These two groups of dynamic PO_2_ profiles, high and low P_min_ data sets, have the same baseline (CR cycles #1–3) and different PO_2_ levels during the “steady-state” contraction (CR cycles #5–9). Restoration of PO_2_ in both groups began immediately after the cessation of stimulation (CR cycles #10–14).

**FIGURE 5:**
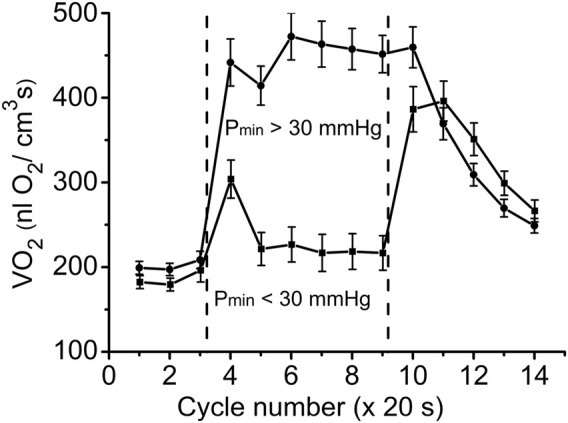
Time courses of muscle VO_2_ during rest, contraction and recovery periods. All experimental data were sorted into two groups according to P_min_. These two groups are also named as “low workload” (P_min_ < 30 mmHg) and “high workload” (P_min_ > 30 mmHg). In both groups the dynamic VO_2_ profiles have the same baseline level at rest (CR cycles #1–3) and different VO_2_ during the “steady-state” contraction (CR #5–9). After cessation of stimulation, VO_2_ in the low workload data set remained high for one CR cycle, and then decreased exponentially. In the high workload group VO_2_ during contraction period was low, but increased rapidly after end of contraction (CR cycles #10–14). Note the statistically significant VO_2_ peak at CR cycle #4, presumably due to utilization of local oxygen storage.

With further analysis, two distinct responses were identified across all applied stimulation frequencies. The VO_2_ responses to 1 and 4 Hz muscle contraction were very different (Figure 3), while the data set for the VO_2_ response to 2 Hz stimulation contained both types of responses. This indicates that, due to the dynamic heterogeneity of the measured microscopic regions of muscle (Poole and Jones, 2012; Heinonen et al., 2015) the responses to stimuli vary at the same stimulation rate. Since the difference in functional response between the data with stimulation at 4 Hz and 1 Hz is due to increased workload, we will assign them to the low workload and the high workload domains. At the same time, data with 2 Hz stimulation exhibit the properties of the first (16 PO_2_ data lines or records) or second type (22 PO_2_ time courses) of functional response. In order to enhance the contrast between VO_2_ responses, all data were sorted into two groups, in which the PO_2_’s during contraction were either above or below 30 mmHg (Figure 4; Figure 5; Table 2). For this purpose the PO_2_ values for the period of sustained contraction (CR cycles #5–9) were averaged and designated as minimal PO_2_, P_min_. Thus, all VO_2_ and PO_2_ curves fall into two groups with P_min_ < 30 and P_min_ > 30 mmHg characterized by two different types of functional response, conditionally named as low P_min_ and high P_min_ data sets, respectively (Table 2; Figure 4; Figure 5). An empirical analysis of the experimental curves gave reason to believe that sorting according to the “30 mmHg criterion” corresponds to a division of the data into low and high workload domains.

**TABLE 2:** Two sets of experimental data PO_2_ and VO_2_ grouped according to average PO_2_ during “steady-state” contraction (CR cycles # five to nine, marked Pmin).

--	Rest		Contraction		Recovery	
P_min_ data sets, (number)	(#1–3) PO_2_ mmHg	(#1–3) VO_2_ nl O_2_/cm^3^s	(#5–9) PO_2_ mmHg	(#5–9) VO_2_ nl O_2_/cm^3^s	(#10–14) PO_2_ τ, s	(#10–14) VO_2_ τ, s
P_min_ >30, (57)	75 ± 1	202 ± 5	46 ± 1	452 ± 11	35	45
P_min_ <30, (48)	70 ± 1	186 ± 6	19 ± 1	220 ± 9	56	*

Data entries are presented as Mean ± SE. The number of averaged experimental curves, i.e., recorded PO_2_ and VO_2_ time courses in individual microscopic regions of muscle, are presented in parentheses in the first column. Transient marked (*) could not be fitted with a monoexponential time course.

Each of three different experimental states of the muscle (rest, contraction and recovery) contains several individual CR cycles. The PO_2_ and VO_2_ for rest (CR cycles #1–3) and “steady-state” contraction (# 5–9) are combined in groups and averaged; thus, the total number of data points in these groups has to be multiplied by 3 and 5, respectively. The last two columns show the time constants for an exponential fit of post-contraction transients of PO_2_ and VO_2_ (CR, cycles #10–14).

These two sets of experimental data have distinct patterns of response to contraction. The baselines for PO_2_ and VO_2_ (CR cycles #1–3) were the same and the onset transients were similarly fast, but the values of oxygen consumption in the contracting muscle (CR cycles #5–9) differed by more than two-fold (*p* < 0.01). At muscle sites with low P_min_ (P_min_ < 30 mmHg) the VO_2_ response started from a VO_2_ peak (CR cycle #4; VO_2_ = 304 ± 22 nL O_2_/cm^3^ s; n = 39; *p* < 0.01) significantly above the following steady state contraction period (CR cycles #5–9; VO_2_ = 220 ± 9 nL O_2_/cm^3^ s; n = 221).

In the post-stimulation period, the PO_2_ started rising immediately, but at different rates in the two groups. Monoexponential PO_2_ restoration occurred much faster in the group of muscle regions with P_min_ > 30 mmHg (Table 2). Excess post-contraction oxygen consumption (EPOC) also occurred quite differently in the two groups of data. The decrease of VO_2_ was significantly slower than that of PO_2_, and it was also slower in the group with P_min_ < 30 mmHg (Table 2; Figure 5). A special feature of the post-contraction VO_2_ transients was the extension of a high VO_2_ level for one CR cycle at muscle regions with P_min_ > 30 mmHg and for two CR cycles at the tissue sites with low P_min_. Thus, in the muscle regions with high P_min_ the VO_2_ remained elevated as during contraction for about 20 s. In the low P_min_ sites the VO_2_ started rising to its maximum level and then remained high for about 40 s before decreasing toward baseline (Figure 5). In both data groups the VO_2_ recovery occurred substantially more slowly than did the restoration of the PO_2_ values (Table 2).

## Discussion

### PO_2_ and VO_2_ at 1, 2 and 4 Hz stimulation

At all stimulation frequencies the onset of contraction evoked a VO_2_ rise and PO_2_ fall, which occurred within the duration of a single CR cycle (20 s). No typical time delay (Hirai et al., 2018; Poole et al., 2020) in the PO_2_ fall was detected, since it was substantially shorter than a single CR cycle.

The magnitude of the PO_2_ drop depended on the workload intensity, i.e., stimulation rate, so that an elevated capillary transmural PO_2_ difference could enhance the oxygen diffusion flux from capillaries. This factor provided a 1.5 times increase in VO_2_ at 1 Hz and a 2.4 times increase at 2 Hz stimulation frequencies. However, this trend did not apply to contraction at 4 Hz, when the VO_2_ was even lower than at 1 Hz stimulation.

The post-contraction increase of PO_2_ started immediately after the cessation of stimulation, although the high VO_2_ was sustained for about one CR cycle for the experimental groups at 1 and 2 Hz stimulation. The post-contraction dynamics of VO_2_ at 4 Hz was different from the transients produced by lower frequencies of stimulation. Upon the completion of 4 Hz stimulation, VO_2_ was maximized during one CR cycle, and then remained at a high level for another CR cycle before starting to decrease (Table 1; Figure 3). Features of transients in interstitial PO_2_ and muscle VO_2_ depended on the intensity of stimulation and contrasted more when comparing groups of data from different workload domains. An alternative interpretation of the post-contraction changes at 4 Hz stimulation is that any increase in O_2_ supply was insufficient to support VO_2_, hence the drop in PO_2_ and increased O_2_ extraction. But by cycles six to nine there was an increase in flow resulting in an increase in PO_2_ and hence an increase in VO_2_ due to VO_2_ dependence on PO_2_.

### Comparison of low and high workload domains

In the groups of data for muscle regions with high and low P_min_ the rest-to-work transition of VO_2_ and PO_2_ occurred within a single CR cycle (Figures 4, 5). That is in good agreement with the characteristic response time for these variables, obtained in experiments on dog and human muscles *in situ* (Grassi et al., 1998; Bangsbo, 2000; Grassi et al., 2002) and isolated muscle fibers (Hogan, 2001), microcirculatory measurements in the rat spinotrapezius muscle (Behnke et al., 2002; Poole et al., 2005; Hirai et al., 2018; Poole et al., 2020) and in the mathematical modeling of oxidative phosphorylation in mitochondria (Spires et al., 2012; Wilson, 2015a).

A more than two-fold increase in oxygen consumption at the rest-to-work transition was achieved in muscle regions with high P_min_, while in the group with low P_min_, a brief VO_2_ increase was followed by a VO_2_ slightly above the resting level (Figure 5; Table 2). In the low workload domain, the increase in O_2_ consumption and interstitial PO_2_ are inversely related. From rest-to-work, VO_2_ increased from 202 to 452 nL O_2_/cm3s, while the PO_2_ decreased from 75 to 46 mmHg. However, this relationship was broken in the data group with high workload. Interstitial PO_2_ decreased from 70 to 19 mmHg, while the average VO_2_ increased from 186 to 220 nL O_2_/cm3s. In this case, VO_2_ increased only at the beginning of the working period, and then returned close to the resting level. The elevated oxygen consumption at the beginning of the contraction period (CR cycle #4) in the low P_min_ data set may serve as evidence of a brief period of oxygen supply supported by local oxygen storage (Figures 3, 5). Previously published data on the rate of oxygen disappearance in this muscle reported the ability of local oxygen storage to supply muscle respiration for about one CR cycle (Golub and Pittman, 2012).

An important finding of this study was the time lag of VO_2_ remaining high after the end of stimulation for about 20–40 s (Figure 5). Interstitial PO_2_ started rising immediately after the end of contraction, almost twice as fast as in the low workload domain (Table 2; Figure 4). In the regions of muscle with high P_min_, the maximal VO_2_ value extended for one CR cycle, followed by a decrease with a time constant of 45 s (Table 2). In the set of data with low P_min_, post-contraction VO_2_ increased nearly two-fold in one CR cycle #10, then remained high in CR cycle #11. The VO_2_ decline began in the next CR cycle #12 at the same slow rate. The post-contraction transients of VO_2_ were slower than those of PO_2_ and no symmetry was observed with the rapid changes at the onset of contraction.

### Analysis of the VO_2_(PO_2_) cyclogram for high and low workload in the muscle

The experimental procedure was carried out cyclically: first, data were obtained at rest, then electrical stimulation was turned on for 6 CR measuring cycles. The muscle was then left at rest for about 10 min to recover before the next CR cycle. It is convenient to display such processes on a cyclogram in which the time parameter is excluded and the data are presented in the form of VO_2_ vs. PO_2_ points. Analysis of the current data was based on a previously published study on the oxygen dependence of tissue respiration (Golub et al., 2018).

The analogy between oxygen tension and oxygen flux in organs, and the voltage and current in electrical circuits is traditionally used for interpretation and mathematical modeling of oxygen transport and its control (Piiper and Scheid, 1975a; Piiper and Scheid, 1975b; Piiper, 1982; Piiper, 1992; Powell and Hempleman, 1993; Piiper and Scheid, 1999; Piiper, 2000; Wagner, 2000; Spires et al., 2013). This analogy is based on the applicability of Ohm’s and Kirchhoff’s laws to a circuit delivering oxygen to parenchymal cells, which is true when properly identifying the corresponding variables. In schematic diagrams the components of O_2_ transport are usually represented by resistor symbols, though conductance, the reciprocal of resistance, is used in the physiological models to avoid confusing it with the hemodynamic resistance in vessels.

**FIGURE 6:**
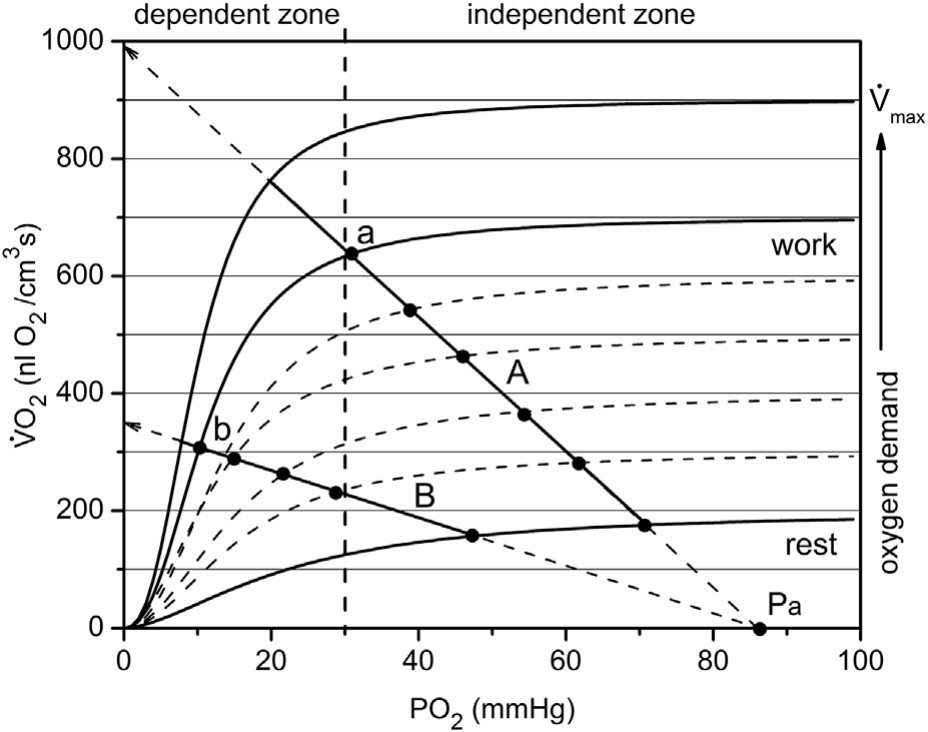
The mechanism of passive balance of oxygen delivery and consumption in skeletal muscle. The dependence of muscle respiration VO_2_ on interstitial PO_2_ is expressed by a sigmoid curve consisting of segments relatively dependent and independent of PO_2_. In addition, the oxygen demand increases with increasing workload, and each level of workload has a separate sigmoid curve. All together they make up a set of characteristics of the oxygen dependence of respiration, separated approximately into O_2_-dependent and -independent zones at a PO_2_ of about 30 mmHg. Oxygen delivery is linearly dependent on the difference in oxygen tension between arterial blood and interstitial space: (Pa—PO_2_). Depending on the conductance Go of the oxygen delivery pathway, the line may go steep or flat, but will cross the point Pa on the abscissa. The point of intersection between the sigmoid curve VO_2_ (PO_2_) and the line (for example, A or B) is the solution to the delivery/consumption balance problem. When the respiration rate of the muscle changes, the point will move along the line. Such sliding within the independent zone is capable of supplying the muscles with oxygen over a wide range of PO_2_. For the less steep O_2_ supply line B, there are restrictions on oxygen consumption due to the supply. Points a and b belong to the same workload curve, but supply lines A and B have different values of O_2_ conductance, Go. Therefore, the muscle in state b performs the same work as in state a, but under conditions of reduced PO_2_. This occurs due to the reserves of oxygen, ATP and phosphocreatine in myocytes, which are replenished due to high VO_2_ following the period of muscle work (Figure 5).

In the electrical analogy of the oxygen supply, oxygen flux V_s_ and respiration rate VO_2_ in myocytes are equivalent to an electrical current. This allows plotting both variables in the same coordinates VO_2_(PO_2_) (Figure 6). Oxygen delivery and consumption are connected in series, so O_2_ flux and consumption are equal (in absolute value) during steady states. For simplicity, we assume that fully oxygenated arterial blood entering the muscle has plasma O_2_ tension Pa. The voltage is represented by the transcapillary difference of oxygen tensions, as between blood and interstitium: (P_a_—PO_2_), where PO_2_ represents interstitial oxygen tension. A coefficient of proportionality between V_s_ and (P_a_—PO_2_) in Ohm’s law is the overall transport conductance, G_o_. The dependence of the oxygen delivery rate on PO_2_ is determined by a straight line passing through the point Pa at zero O_2_ consumption (Figure 6, lines A and B):

$$V_{s}=G_{o}\left(P_{a}-PO_{2}\right)$$ (1)

In this equation, Vs. represents the oxygen flux or rate of oxygen delivery, driven by the difference of arterial oxygen tension and interstitial PO_2_ through the conductance Go (Figure 6). Data from this study used for rat arterial blood PO_2_ was P_a_ = 94 mmHg (Turek et al., 1978). At PO_2_ = 0, this line intersects the vertical axis for Vs. and VO_2_ at the point V_s_ = Go·Pa, from which it follows that Go is equal to the slope of the line that determines the rate of oxygen delivery. A larger oxygen conductance (Figure 6: line A vs. line B) corresponds to a greater efficiency of oxygen transport (Piiper and Scheid, 1975b; Piiper, 1982; Piiper, 1992).

It should be noted that the overall conductance, Go, is determined jointly by the convective and diffusive conductances according to the rule:

$$G_{o}=\frac{G_{c}∙G_{d}}{G_{c}+G_{d}}$$ (2)

Diffusive conductance G_d_ is determined by the stable anatomical structure of the microvasculature and a relatively constant density of perfused capillaries at different workloads (Turek et al., 1978; Piiper, 1992; Piiper and Scheid, 1999). Thus, the changes in overall oxygen conductance are mainly determined by variation of the convective O_2_ conductance G_c_.

The analysis of the factors involved in the balance between O_2_ supply and demand at different levels of metabolic activity tend to neglect the role of the PO_2_ dependence of tissue respiration rate (Piiper and Scheid, 1975b; Piiper and Scheid, 1999; Wagner, 2011a). Recent measurements (Golub and Pittman, 2012; Wilson et al., 2012; Wilson et al., 2014; Wilson, 2015a; Golub et al., 2018) have brought evidence that the respiration rate of cells is PO_2_-dependent and that at different levels of functional activity these dependencies are similar in shape but different in amplitude. The oxygen dependence of respiration is a combined effect of metabolic regulation in mitochondria (Wilson et al., 1977; Wilson et al., 2012; Wilson, 2015a; Wilson, 2015b; Wilson, 2016) and the intracellular diffusion barrier (Golub and Pittman, 2012). Each curve of this type is Ohm’s Law for the cell, and each level of energy expenditure has a separate curve defined by workload.

The oxygen dependence of the respiratory rates on interstitial PO_2_ in this muscle were obtained previously (Golub et al., 2018). These dependences are expressed as a set of sigmoidal curves VO_2_ (PO_2_), with magnitude directly dependent on workload (Figure 6). If lines A and B in Figure 6 represent oxygen flux to the muscle (according to Eq. 1) and the sigmoid line represents the O_2_ consumption rate, then the intersection point corresponds to the equality of oxygen supply and consumption. For each level of workload, there is only one equilibrium point that belongs to both the characteristic curve of tissue oxygen supply, V_s_, and oxygen consumption rate, VO_2_. For each level of workload, there is only one equilibrium point that belongs to both the characteristic curve of myocyte respiration VO_2_ (PO_2_) and the line for the rate of O_2_ supply V_s_ (PO_2_) described by Eq. 1.

Each experimental point is determined by a pair of values in the coordinates VO_2_ vs. PO_2_. These data are shown separately in Figure 4 and Figure 5. At a given PO_2_, the muscle receives an oxygen flux V_s_ and consumes O_2_ at the rate VO_2_. Thus, each state of muscle respiration corresponds to the conductance G_o_, which can be determined as:

$$G_{o}=\frac{{VO}_{2}}{P_{a}-{PO}_{2}}$$ (3)

This expression is valid for stationary states represented by CR #1–3 (rest) and CR #5–9 (work). Eq. 3 can also be applied to transients, provided the influence of local oxygen storage is negligible. Data used for rat arterial blood PO_2_ was P_a_ = 94 mmHg (Turek et al., 1978).

**FIGURE 7:**
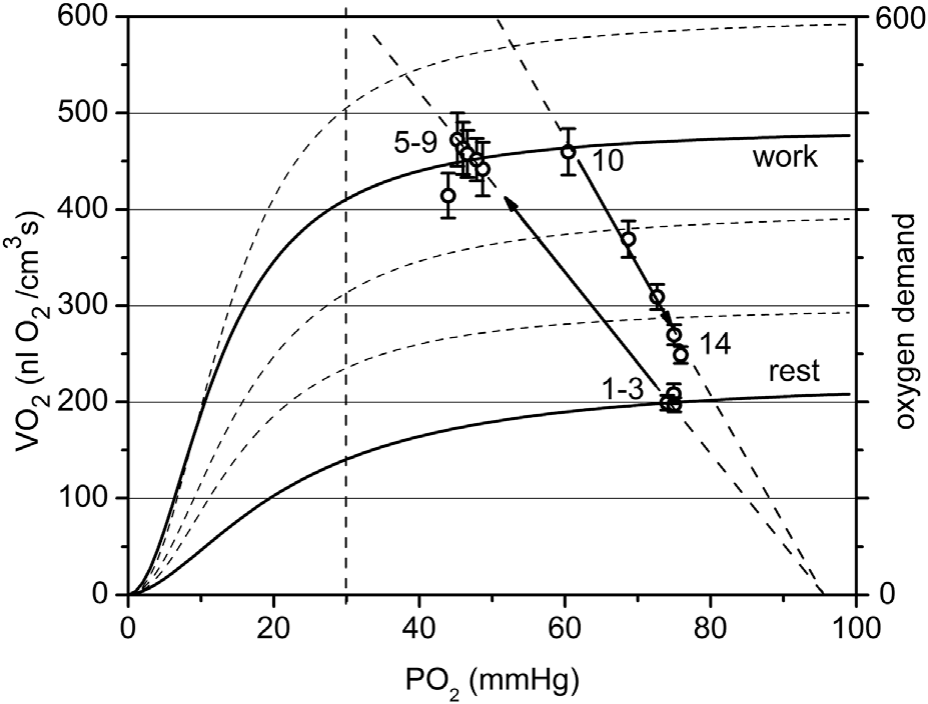
The cyclogram (points #1–14) represents in coordinates of VO_2_ vs. PO_2_ the complete experimental cycle for the low workload data group: stationary rest (points one to three), rest-to-work transition, stationary work (points five to nine), work-to-rest transition (points 10–14). The calculated conductance for the steady state is 10.6 at rest and 9.4 nL O_2_/(cm^3^·s·mmHg at work. The rest-to-work transition line has a conductivity of 9.5, close to that in steady work, but lower than at rest. The reverse pathway or work-to-rest runs along the line with increased O_2_ conductance Go = 13.7. The vertical dashed line separates the relatively dependent and independent zones for VO_2_(PO_2_) curves).

**FIGURE 8:**
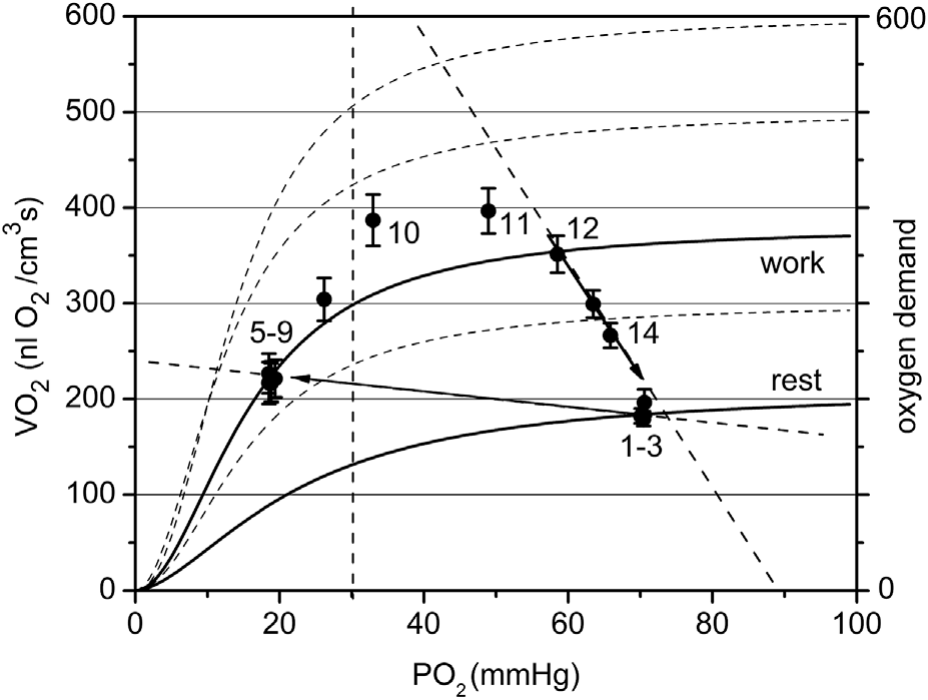
The cyclogram (points #1–14) represents the complete experimental cycle for the high workload data group: stationary rest (points one to three), rest-to-work transition, stationary work (points five to nine), work-to-rest transition (points 10–14). The calculated O_2_ conductance for the steady state is 7.8 at rest and 2.9 nL O_2_/(cm^3^·s mmHg) at work. The position of the “steady work” data points #five to nine in the dependent zone is characterized by a low respiration rate, forced by a low PO_2_. The rest-to-work transition line goes beyond the scope of the model, since it does not pass through the point P_a_. The proposed reason for this behavior is the development of tetanus, which dramatically reduces oxygen conductivity by reducing blood flow in the muscle. The return to the state of rest follows a wide arc with a long-term increase in the respiration rate and then its decrease along the supply line with G_o_ about 13.4 nL O_2_/(cm^3^·s mmHg).

The graphical analysis was applied to two contrasting groups of experimental data for low and high workload (Figure 7; Figure 8). These two groups of VO_2_(PO_2_) data points for oxygen supply V_s_ and consumption rate VO_2_ are plotted over the set of sigmoidal curves representing the oxygen dependence of respiration for this muscle (Golub et al., 2018). The location of the points for both resting data groups is the same, but the difference is revealed in transitions from rest (CR points one to three) to steady state contraction (CR points five to nine). The rest-to-work transition defines a corresponding O_2_ supply line, which crosses the PO_2_ axis at P_a_ (Figure 7 and partly in Figure 8).

In the low workload data set (Figure 7) a steady state conductance, calculated from Table 2, at rest was G_o_ = 10.6, while at work it was 9.4 nL O_2_/(cm^3^s·mmHg). The value at rest is the starting point of the cycle, with a minimum flow of oxygen through the muscle. In steady state muscular work, the conductance is moderately reduced, presumably proportional to the time fraction of muscle contraction, or fill factor. The rest-to-work transition going along the O_2_ supply line with G_o_ = 9.5 nL O_2_/(cm^3^s·mmHg), was close to the value of G_o_ for the steady state at work. This line is entirely located in the PO_2_-independent segments of the VO_2_(PO_2_) characteristics. This indicates that the contracting muscle (CR points five to nine) had an enhanced O_2_ supply mostly due to the increased PO_2_ gradient between blood and muscle cells. Following the cessation of stimuli, the overall O_2_ conductance instantly increased to 13.7 nL O_2_/(cm^3^s·mmHg) during the return to the resting state (CR points 10–14). An increase in conductance during the work-to-rest transition reflects the development of vasodilation in the muscle (Marshall and Tandon, 1984). The changes in G_o_ resulted in a narrow loop trajectory describing the complete cycle of muscle contraction/relaxation.

The data set collected at high workload (P_min_ < 30 mmHg) is displayed in Figure 8. With a high workload, the difference in overall conductance G_o_ at rest and work is more pronounced: 7.8 and 2.9 nL O_2_/(cm^3^s·mmHg), respectively. The conductance at the starting point is noticeably lower than that in the low workload domain and conductance during steady work dropped substantially. We hypothesize that this is due to the impact of muscle contraction at this stimulation rate on local microvascular perfusion, which greatly increased the fill factor for the contracted muscle phase. The O_2_ supply line for the rest-to-work transition was directed into the PO_2_-dependent zone of the VO_2_(PO_2_) characteristics for myocytes and the O_2_ supply line for transition is near horizontal, with a low G_o_ = 0.8 nL O_2_/(cm^3^s·mmHg). The cause of this phenomenon lies in the limitation of blood flow in an intensely contracting muscle. In the classic experiments of Barcroft (Barcroft, 1972a; Barcroft, 1972b), it was established that static muscle contraction at a workload higher than 20% of the maximum level causes the hindrance and arrest of blood flow. The cause for blood flow arrest is the supra-systolic tissue pressure developed in a contracting muscle, demonstrated by direct measurements (Styf et al., 1987; Crenshaw et al., 1992; Hargens et al., 1992). The hindrance of blood flow in muscle was detected at a compartment pressure above 30–80 mmHg (Jarvholm et al., 1988; Crenshaw et al., 1992). In a rhythmically contracting muscle the intramuscular pressure has a series of peaks, so that the mean time of the arrested blood flow may be less than 50% of the total time (Hargens and Ballard, 1995; Styf et al., 1995; Saltin et al., 1998).

Other potential explanations for the stratification of the data into two groups according to P_min_ include heterogeneity in capillary density, geometry, and capillary RBC supply rate between different regions. In addition, the relative placement of the measurement region in terms of arteriolar or venular end of capillaries and A-V units could also influence the measured interstitial PO_2_s due to longitudinal SO_2_ gradients along the length of capillaries. This may explain why some regions at 2 Hz contraction frequency fell within the P_min_ < 30 mmHg bin when no physical impediment to vascular filling was present. Another possibility is that some regions have relatively lower RBC supply/tissue volume, which is somewhat supported by the lower resting PO_2_ in the high workload group—while one might expect lower perfused regions to increase RBC supply proportionally to increases elsewhere, it is possible that more reactive regions, in terms of arteriolar vasodilation, could shift the distribution of blood flow to regions with higher conductance.

After the cessation of contraction in the high workload data group (Figure 8), the transition from work-to-rest occurred in a wide arc, with high respiration rate and rapidly rising PO_2_. The final segment of the cycle runs along the line with approximately G_o_ = 13.4 nL O_2_/(cm^3^s·mmHg), a much higher conductance than in the steady state at rest.

**FIGURE 9:**
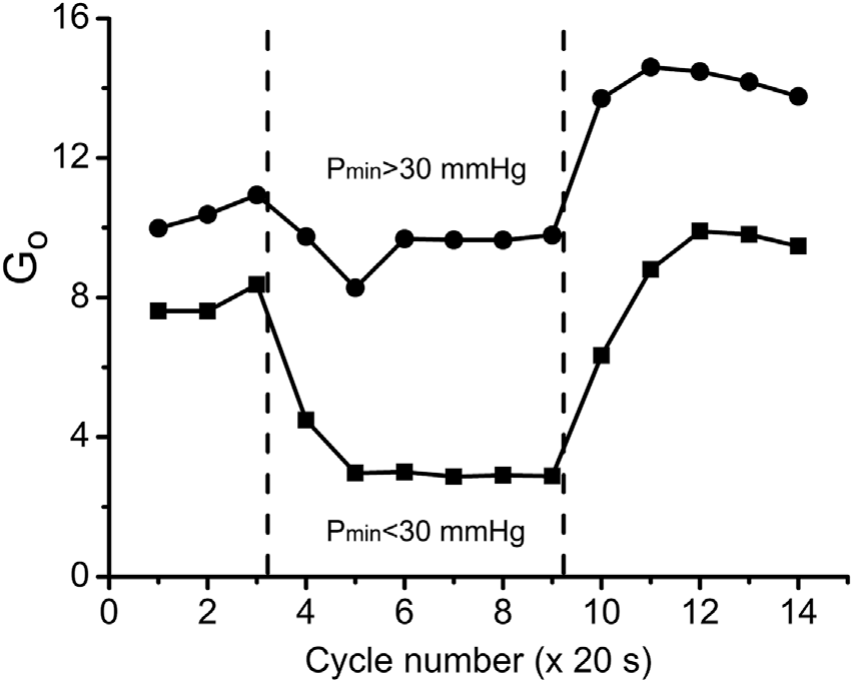
Overall oxygen conductance G_o_ (units: nl O_2_/cm^3^ s mmHg) in the two workload groups of data during the transitions through three experimental states: rest, work and recovery. G_o_ values were calculated according to Eq. 3 using data from Figures 5, 6. Muscle regions with low workload (high P_min_) during the contraction kept G_o_ = 9.4 at almost the same level as at rest G_o_ = 10.6, but there was an increase of G_o_ during the recovery phase, indicating vasodilation. In the data group of high workload (low P_min_) G_o_ was reduced from rest G_o_ = 7.8 to contraction G_o_ = 2.9, presumably due to high intramuscular pressure, developed by intensive muscle contraction. Following the cessation of stimulation, G_o_ reached a peak value which was lower than in the low workload data group.

The employment of the conductance parameter G_o_ to describe steady states and transient processes in muscle makes it possible to represent the cycle of oxygen delivery/consumption during a full cycle of work and rest (Figure 9). The difference between G_o_ in the two data groups was large during the steady rest and contraction periods and remained large in the post-contraction G_o_ transient. The post-contraction time lag of VO_2_ had no effect on the G_o_ transient that started at the end of the contraction period (Figure 9). G_o_ had reached a maximum at 40–60 s after the end of stimulation and then started to decrease. The analysis of G_o_ demonstrated that no active hyperemia was developed during isometric contractions at high workload (P_min_ < 30 mmHg); but oppositely, that type of contraction was accompanied by limited blood flow. Blood flow restoration and hyperemia occurred during the post-contraction period, contributing to the formation of the PO_2_ and VO_2_ transients (Figures 5, 6). Note that G_o_ for stationary rest and work is an exact value, but the G_o_ for transients can be distorted by the effect of local oxygen storage.

By comparing the time courses of G_o_ during the rest-work-rest cycle at low and high workload we distinguish two types of changes in the state of delivery/consumption of oxygen in the muscle. At low workload, the rest-to-work transition occurs with a slight decrease in oxygen conductivity, and the reverse transition is associated with an increase in conductivity above the rest level. Under high workload, steady work occurs under severely limited delivery conditions. At the same time, myocytes are in the region of respiration dependent on PO_2_, which greatly limits their respiration rate. Restoration of muscle resources begins after the end of steady work with the transition to the zone of VO_2_, which is virtually independent of PO_2_. Recovery is characterized by a long period of high PO_2_ and VO_2_ with a return to rest along a line with high conductance. The changes of G_o_ during rest, contraction and recovery agree with classical experimental evidence that contractions engender a mechanical impediment to the passage of blood through human muscle and the mechanical hindrance of contraction reduces blood flow (Folkow et al., 1969; Barcroft, 1972a; Barcroft, 1972b; Chilian and Marcus, 1982; Styf et al., 1987; Jarvholm et al., 1988) and leads to the development of vasodilation after the end of the work period (Marshall and Tandon, 1984).

## Conclusion

The mechanisms responsible for the coordination of functional activities by the musculature and vasculature in skeletal muscle, over a wide range of workloads, remain a problem of interest in physiology. A dominating paradigm in muscle physiology is that the VO_2_ kinetics associated with muscle contraction is principally determined by the activity of mitochondria in myocytes, rather than by the integrated O_2_ transport system. This theory was rooted in the belief that cellular respiration is independent of oxygen level. New evidence regarding the oxygen dependence of respiration requires a reevaluation of the role of interstitial PO_2_, which is not only a gauge of the balance between oxygen delivery and consumption, but also a modulator of tissue respiration (Golub et al., 2018).

Simultaneous measurements of PO_2_ and VO_2_ were conducted in the muscle interstitial space during periods of rest, isometric contraction and recovery. Increasing the intensity of contractions from 1 to 4 Hz also increased the extent of muscle regions with a contraction-induced fall of PO_2_ below 30 mmHg, which was accompanied by a reduced VO_2_ due to limitation in O_2_ delivery. Тhe results indicate that in the range of stimulation frequencies from 1 to 4 Hz, there is a transition from a series of twitches to a contraction, which impedes the delivery of oxygen to myocytes.

Two types of functional response were distinguished in contracting muscle, depending on the effect of the increased workload on steady-state PO_2_. In muscle regions where interstitial PO_2_ remained above 30 mmHg during steady contraction (low workload), VO_2_ increased rapidly and stabilized at a high level. Total O_2_ conductance remained almost the same as at rest. After the end of the contraction period, the PO_2_ increased immediately, while high VO_2_ lagged behind for 20 s and then slowly decreased. At a high workload the PO_2_ at contraction fell below 30 mmHg, the VO_2_ was limited by O_2_ delivery almost to its resting level. Because of the strong oxygen dependence of respiration below 30 mmHg, that level of interstitial PO_2_ and VO_2_ may be considered as evidence of low oxygen conductance due to mechanical hindrance to blood flow.

Upon cessation of 4 Hz contraction, the low VO_2_ sharply increased to a high level, then remained elevated for 40 s before starting to fall at a much slower rate than in low intensity contractions. The post-contraction transients of PO_2_ and VO_2_ were not synchronous and had different time constants, all much slower than the onset transients.

Complex trajectories of the process of restoring the oxygen balance after work indicate a dependence on other variables, except for VO_2_ and PO_2_, as is in the case of steady states. First of all, this is the convective conductance of oxygen, which can decrease due to intramuscular pressure or increase due to vasodilation. Another factor is intramuscular oxygen reserves, which are not involved in steady states, but can be depleted or replenished with oxygen during transients.

In contracting muscle, the balance between oxygen delivery and consumption, known as an “adequate O_2_ supply,” is violated during transients. The new balance may not be achievable if the muscle workload exceeds the limit imposed by the increase in blood flow. Consequently, under intense muscle contraction there is a mismatch between oxygen supply and demand, which can be compensated during the post-exercise recovery by the development of hyperemia. Cellular respiration and the microcirculation in the muscle are integrated into a system of oxygen processing, capable of complex adaptive behavior that includes time separation between work and compensation for energy expenditure.

## Data Availability

The original contributions presented in the study are included in the article/supplementary material, further inquiries can be directed to the corresponding author.

## Abbreviations

PO_2_, oxygen tension in the interstitium; VO_2_, oxygen consumption (equal to O_2_ flux); PQM, phosphorescence quenching method (or microscopy); CR, compression and recovery cycle for VO_2_ measurement; ODC, oxygen disappearance curve.
